# Investigation of protein-protein interactions and hot spot region between PD-1 and PD-L1 by fragment molecular orbital method

**DOI:** 10.1038/s41598-019-53216-z

**Published:** 2019-11-13

**Authors:** Hocheol Lim, Jungho Chun, Xuemei Jin, Jongwan Kim, JeongHyeok Yoon, Kyoung Tai No

**Affiliations:** 1 Department of Biotechnology, Yonsei University, Seoul, Republic of Korea; 2 Bioinformatics and Molecular Design Research Center (BMDRC), Yonsei University, Seoul, Republic of Korea; 3 Pharos I&BT Co., Ltd., Anyang-si, Gyeonggi-do, 14059 Republic of Korea

**Keywords:** Quantum chemistry, Protein analysis

## Abstract

Inhibitors to interfere protein-protein interactions (PPI) between programmed cell death 1 (PD-1) and programmed death ligand-1 (PD-L1) block evasion of cancers from immune surveillance. Analyzing hot spot residues in PPI is important for small-molecule drug development. In order to find out hot spots on PPI interface in PD-1/PD-L1 complex, we analyzed PPI in PD-1/PD-L1 with a new analysis method, 3-dimensional scattered pair interactions energies (3D-SPIEs), which assorts significant interactions with fragment molecular orbital (FMO) method. By additionally analyzing PPI in PD-1/antibody and PD-L1/antibody complexes, and small-ligand interactions in PD-L1/peptide and PD-L1/small-molecule complexes, we narrowed down the hot spot region with 3D-SPIEs-based interaction map, which integrates PPI and small-ligand interactions. Based on the map, there are two hot spot regions in PPI of PD-1/PD-L1 and the first hot spot region is important for inhibitors. In particular, _L_Y56, _L_E58, and _L_N66 in the first hot spot of PD-L1 are important for PD-L1-antibodies and small-inhibitors in common, while _L_M115 is important for small-inhibitors. Therefore, the 3D-SPIEs-based map would provide valuable information for designing new small-molecule inhibitors to inhibit PPI of PD-1/PD-L1 and the FMO/3D-SPIEs method provides an effectual tool to understand PPI and integrate PPI and small-ligand interactions at a quantum mechanical level.

## Introduction

Programmed cell death protein 1 (PD-1) is a type I transmembrane receptor belonging to the CD28 family^1^. PD-1 is transcriptionally expressed on activated T cells, B cells and myeloid cells^2–5^. An activated T cell expresses PD-1 on its surface and produces interferons which can induce many tissues to express programmed death ligand-1 (PD-L1)^6,7^. PD-1 binds to its ligand to deliver negative or co-inhibitory signals^1,8^, and results in the activation of inhibitory kinases related with proliferation and adhesion of T cells^1^. The PD-1/PD-L1 pathway downregulates immune responses and preserves tolerance to self-antigens by curbing improper autoimmune responses under normal physiological conditions^5,9^. PD-1 ligands are commonly upregulated in different tumor types, where they are used to evade immune responses^1,7^. Blocking the PD-1/PD-L1 interactions can reanimate exhausted T cell phenotype and result in normalization of antitumor response^7,10^. The success in clinical studies, where anti-PD-1 agents reduced the remote metastasis of melanoma cells or colon cancer cells, improved the knowledge of PD-1/PD-L1 as an important anticancer target in human beings^8^. Although several nonpeptidic small-molecule inhibitors of the PD-1/PD-L1 interaction are reported^11^, it still has been challenges to develop small molecular inhibitors targeting PD-1 and PD-L1 due to their flat and large interface. The lack of information about protein-protein interactions (PPI) between PD-1 and PD-L1 has hindered the anti-PD-1-targeted, or anti-PD-L1-targeted, small molecular drug development in cancer therapies.

Protein-protein interactions (PPI) play a crucial role in the regulation of many biological processes and a functional disorder of PPI strongly results in a variety of diseases including cancers^12^. However, PPI-targeted small molecular drug development seemed intractable due to relatively flatter and featureless interfaces of PPI, compared to traditional pharmacophore based targets^13^. Encouraged by successful assistances of the computational methods in drug discovery, there have been many attempts to develop PPI-targeted drugs by using in silico approaches based on the molecular mechanics (MM) methods^14,15^. Nonetheless, a direct application of the present MM to PPI has limits, because MM methods depend on predetermined parameters optimized to reproduce average values and classical mechanics could not describe the molecular system precisely. The use of quantum mechanics (QM) comes to the fore, because QM could overcome the limitations of MM. Although QM could not be easily applied to the large biological systems, such as PPI, due to huge computational cost^15^, it provides the analysis of the components of molecular interaction in post Hartree-Fock level with energy decomposition analysis (EDA) method, developed by Morokuma *et al*.^16^. The EDA in QM can describe the important dispersion interactions and non-classical interactions, including CH-π, cation-π, and so on in PPI, which were not easy for MM to describe.

As a practical application of EDA, Kitaura *et al*. developed fragment molecular orbital (FMO) method for large molecules and molecular systems in 1999^17^, and pair interaction energy decomposition analysis (PIEDA) in 2007^18^. It has advantages of drastically reducing the computing time without compromising the accuracy of the results compared with traditional QM method, and of providing PIEDA as a practical application of EDA. Recent progress in method development and applications was well summarized in review articles of Fedorov *et al*.^19,20^ It has been successfully applied to investigate the protein-ligand interactions based on PIEDA qualitatively and quantitatively^21,22^. In case of PPI as a large molecular system, there have been a few cases to apply FMO method, where a partner protein was defined as a fragment for facilitating PPI analysis and it required huge computational cost^23^.

In this work, we applied FMO method to investigate PPI between PD-1 and PD-L1. In order to find hot spot residues in PD-1/PD-L1 complex, we performed FMO calculation of wild type complex. For analyzing PPI with computational cost being low by using a-fragment-per-a-residue fragmentation scheme, we devised a 3-dimensional scattered pair interaction energies (3D-SPIEs) analysis tool. The 3D-SPIEs method sorts pair interaction energy (PIE) out by using distance between fragments and energy value information, so it provides a 3-dimensional scatter plot and important interactions in PPI. To narrow down hot spot region in PD-1/PD-L1, we performed FMO calculations of PD-1/antibody complexes, PD-L1/antibody complexes, PD-L1/peptide complexes, and PD-L1/small-molecule complexes. For integrating PPI in the antibody complexes and the small-ligand interactions in small-ligand complexes, we made 3D-SPIEs-based interaction map in PD-1/PD-L1. In order to validate PPI predictability of the FMO results, we compared them with the reported site-directed mutagenesis results. Finally, we summarized FMO/3D-SPIEs results as an interaction map, by integrating the information from PPI and small-ligand interactions, and found the hot spot region in PD-1/PD-L1 at a quantum-mechanical level.

## Results

All X-ray structures in this work are summarized in Table 1. We used subscript notation ‘L’ before amino acid to indicate PD-L1, subscripts ‘HC’ and ‘LC’ to indicate heavy chain and light chain in antibody, and superscripts, ‘A’, ‘B’, ‘C’, ‘G’, ‘H’, and ‘L’, after water molecules to indicate chain arrangement.

**Table 1:** Summary of 14 crystal structures in this study.

Class	Complex	PDB ID	Resolution (Å)
wild-type	PD-1/PD-L1	4ZQK	2.45
PD-1-Antibody	PD-1/Nivolumab	5WT9	2.401
	PD-1/Pembrolizumab	5B8C	2.146
PD-L1-Antibody	PD-L1/BMS-936559	5GGT	2.8
	PD-L1/Avelumab	5GRJ	3.206
	PD-L1/KN035	5JDS	1.7
	PD-L1/Atezolizumab	5XXY	2.9
	PD-L1Durvalumab	5X8M	2.661
peptide	PD-L1/peptide-71	5O45	0.99
	PD-L1/peptide-57	5O4Y	2.3
small molecule	PD-L1/BMS-8	5J8O	2.3
	PD-L1/BMS-37	5N2D	2.35
	PD-L1/BMS-200	5N2F	1.7
	PD-L1/BMS-202	5J89	2.2

### 3-dimensional scattered pair interaction energies (3D-SPIEs)

**Figure 1:**
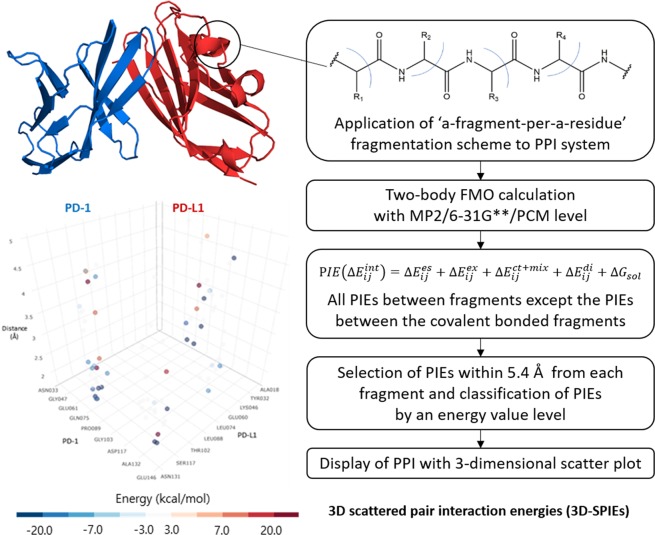
Workflow of 3D scattered pair interaction energies (3D-SPIEs).

Analyzing protein-protein interactions (PPI) between a target protein and a partner protein can be correlated with analyzing pair interaction energy (PIE) in FMO calculations with a-fragment-per-a-residue fragmentation scheme. Because the calculated PIE often show unphysically large values primarily due to electrostatic interactions^24^, we used polarizable continuum model (PCM) for solvent screening effects and introduced distance information to sort out the meaningful PIEs from overestimated PIEs. As for distance information, we selected only PIEs within a specific distance (5.4 Å) between two fragments, which reflected the distance used for the approximate of electrostatic potential in FMO method^25^. The brief scheme of 3-dimensional scattered pair interaction energies (3D-SPIEs) method is shown in Fig. 1.

### Two main hot spot regions in wild-type PD-1/PD-L1 complex

In order to find out amino acids on PPI interface that form hot spots in wild type PD-1/PD-L1 complex, we performed FMO calculation of the wild-type complex and analyzed it with 3D-SPIEs method. It detected 28 PPI between PD-1 and PD-L1, which are summarized in Supplementary Table S1, and 5 water-bridge interactions, which are summarized in Supplementary Table S2. The FMO results were in agreement with the previously reported 13 PPI and 3 water-bridge-interactions of Zak *et al*.^7^ 13 PPI: Y68:_L_D122, Q75:_L_D26, Q75:_L_R125, A132:_L_Q66, I134:_L_Y56, I134:_L_E58, I134:_L_Y123, E136:_L_R113, E136:_L_Y123, E136:_L_R125, N66:_L_D122, Q75:_L_I126, and K78:_L_T20, and 3 water-bridge-interactions: I134:HOH202:_L_Y56, I134:HOH202:_L_E58, and N66:HOH203:_L_A121.

**Figure 2:**
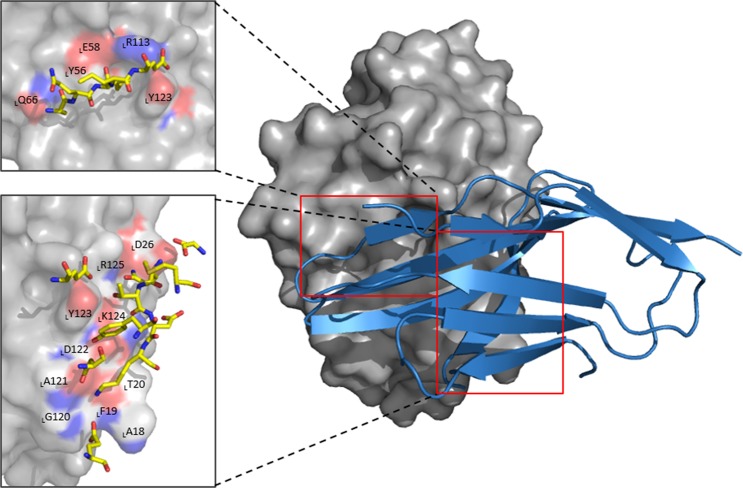
Two main binding sites between wild-type PD-1 and PD-L1. The first binding site in PD-1 consists of A132-E136 and its corresponding site in PD-L1 is comprised of _L_Y56, _L_E58, _L_Q66, _L_R113 and _L_Y123. (left red box) The second binding region in PD-1 includes N66, Y68, M70, S73, N74-K78 and E84. Its corresponding region in PD-L1 has _L_A18-_L_T20, _L_D26 and _L_G120-_L_R125. (right red box) PD-1 and PD-L1 are represented by blue cartoon and gray surface, respectively. The carbon atoms of PD-1 are shown in yellow. In both sticks and surface, nitrogen atoms are colored blue and oxygen in red. All interactions shown here have attractive PIE value more stable than −3.0 kcal/mol, whose magnitudes are ignored.

Zak *et al*. reported three hot spots on the PPI interface between PD-L1 and PD-1^7^ the first hot spot was _L_Y56, _L_E58, _L_R113, _L_M115, and _L_Y123; the second was _L_M115, _L_A121, and _L_Y123; and the third was from _L_D122 to _L_R125. PPI in PD-1/PD-L1 complex analyzed with FMO method are delineated in Fig. 2. In the FMO results, there were only two hot spots on the PPI interface. In the second hot spot of Zak *et al*.^7^, only E136:_L_Y123 were strong, which can belong to the third hot spot. Therefore, we rearranged the reported three hot spot regions into two hot spot regions in PD-1/PD-L1 complex based on the FMO results. (see Fig. 2) The first hot spot region of PD-1 was comprised of residues from A132 to E136 located in the front sheet of PD-1, which corresponds to the first hot spot region of PD-L1, consisting of _L_Y56, _L_E58, _L_Q66, _L_R113, and _L_Y123 located in the opposite β strands to the front sheet of PD-1. The second hot spot region of PD-1 includes N66, Y68, M70, S73–K78, and E84, which corresponds to the second hot spot region of PD-L1, consisting of _L_A18–_L_T20, _L_D26, and _L_G120–_L_I126.

### Hot spot region which two PD-1-antibodies occupied in PD-1

In order to narrow down hot spot regions in PD-1/PD-L1 complex, we performed FMO calculations of two antibody complexes in complex with PD-1 and analyzed them with 3D-SPIEs method. There were the reported antibody complexes in complex with PD-1: nivolumab and pembrolizumab. Although it was reported that nivolumab binds to a different area, compared to pembrolizumab^26^, two antibodies can inhibit PPI between PD-1 and PD-L1, which reflects that there may be the common hot spot area on PD-1 among two PD-1-antibodies and PD-L1.

#### PD-1/Nivolumab

In PD-1/nivolumab complex, the FMO results detected 29 PPI, which are summarized in Supplementary Table S3, and 19 water-bridge interactions, which are summarized in Supplementary Table S4. The FMO results were in agreement with the previously reported 11 PPI of Tan *et al*.^26^. L25:_HC_W52, L25:_HC_K57, D29:_HC_G33, R30:_HC_S30, R30:_HC_N31, K131:_HC_D100, K131:_HC_D101, A132:_LC_Y49, R30:_HC_S32, P130:_LC_T56, and K131:_LC_T56.

The FMO results can provide not only PPI, but also intra-protein interactions (non-PPI) in the single protein. It was reported that there were four potential N-linked glycosylation sites in PD-1 (N49, N58, N74, and N116)^26^. Although they did not interact with nivolumab directly, the mutations of N49A, N58A, N74A, and N116A made binding affinity decrease 1.54, 2.4, 1.58, and 1.12–fold, respectively^26^. The loss of binding affinity may be caused by the change of non-PPI in PD-1, which contributed to PPI. The FMO results detected several non-PPI in the four N-linked glycosylation sites. Because N49 interacted with G47 (−3.792 kcal/mol), V111 (−4.463 kcal/mol), R112 (−23.305 kcal/mol), A113 (−16.251 kcal/mol), and R114 (−8.929 kcal/mol) in PD-1, N58A mutation would change intra-protein interactions in β-sheet 2 (V64) and β-sheet 4 (Q99). Because N-glucan on N58 and N58 interacted with E61 (−41.732 kcal/mol), V64 (−9.327 kcal/mol), P83 (−3.645 kcal/mol), Q99 (−4.843 kcal/mol), and P101 (−4.458 kcal/mol), N49A mutation would change intra-protein interactions in the loop. Because N74 interacted with P72 (−17.496 kcal/mol) and N116 interacted with S118 (−15.175 kcal/mol), the N74A and N116A mutations would change intra-protein interactions in β-turn and α-helix 9, respectively.

#### PD-1/Pembrolizumab

In PD-1/pembrolizumab complex, the FMO results detected 37 PPI, which are summarized in Supplementary Table S5, and 18 water-bridge interactions, which are summarized in Supplementary Table S6. The FMO results were in agreement with the previously reported 14 PPI of Horita *et al*.^27^ and 10 PPI of the traditional quantum chemistry results^28^. 14 PPI: E61:_LC_Y57, N66:_HC_R102, Q75:_HC_T30, T76:_HC_Y101, D77:_HC_S54, D77:_HC_N55, K78:_HC_Y33, K78:_HC_Y101, D85:_HC_R99, R86:_LC_D97, Q88:_HC_N59, E61:_LC_Y34, S62:_LC_Y57, and Q88:_HC_Y35, and 10 PPI: V64:_HC_F103, N66:_HC_F103, Y68:_HC_R102, K78:_HC_R102, K78:_HC_F103, D85:_HC_R102, Q88:_LC_Y33, P89:_LC_Y35, I126:_HC_R102, and I134:_HC_R102.

#### The common hot spot region in PD-1 against two PD-1-antibodies and PD-L1

**Figure 3:**
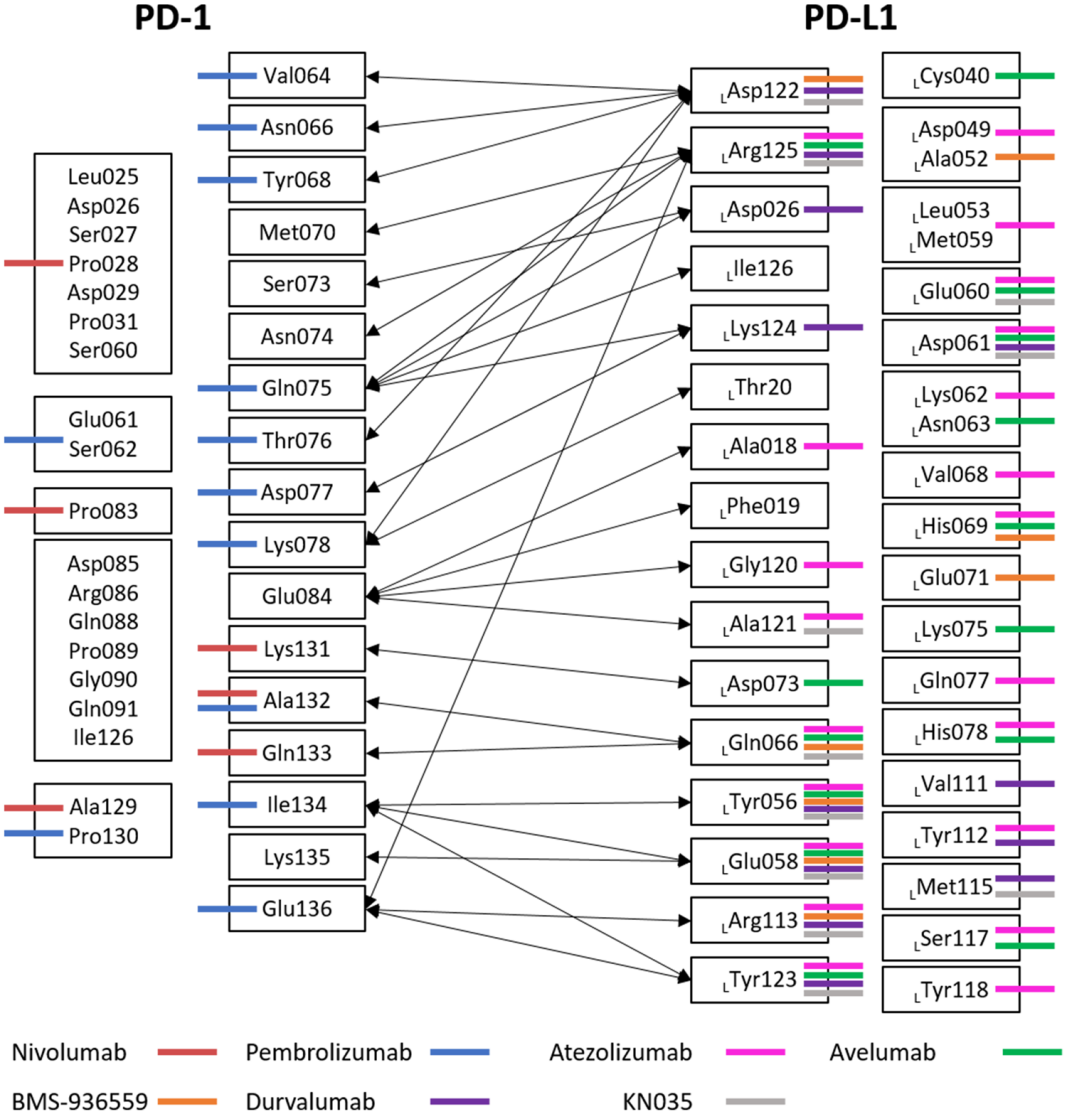
3D-SPIEs-based interaction map in PD-1, PD-L1, and antibodies. PD-1/PD-1-antibodies interactions are shown in the left-hand (nivolumab and pembrolizumab), while PD-L1/PD-L1-antibodies interactions are in the right-hand (atezolizumab, avelumab, BMS-936559, durvalumab and KN035) In the middle, PD-1 and PD-L1 interactions are shown with the black arrows. All interactions shown in this map have attractive PIE value more stable than −3.0 kcal/mol, whose magnitudes are ignored.

In order to investigate the common hot spot regions among two PD-1-antibodies and PD-L1, we illustrate the FMO results in Fig. 3. The common interactions between nivolumab and PD-L1 were K131, A132, and Q133; the common interactions between pembrolizumab and PD-L1 were V64, N66, Y68, N75, T76, D77, K78, A132, I134, and E136; and the common interactions between nivolumab and pembrolizumab were A129, P130, and A132. Although nivolumab had different PPI with PD-1, compared to PPI of pembrolizumab and PD-L1, nivolumab hindered the first hot spot region of PD-L1 through K131, A132, and Q133, and pembrolizumab hindered the region through A132, I134, and E136.

### Hot spot region which five PD-L1-antibodies occupied in PD-L1

In order to narrow down hot spot regions in PD-1/PD-L1 complex, we performed FMO calculations of five antibody complexes in complex with PD-L1 and analyzed them with 3D-SPIEs method. There were the reported five antibody complexes in complex with PD-L1: BMS-936559, Avelumab, KN035, Atezolizumab, and Durvalumb.

#### PD-L1/BMS-936559 (PDB ID: 5GGT)

In PD-L1/BMS-936559 complex, the FMO results detected 17 PPI, which are summarized in Supplementary Table S7. The FMO results were in agreement with the previously reported 5 PPI of Lee *et al*.^29^_L_D49:_HC_G105, _L_D49:_LC_Y32, _L_Y56:_HC_H59, _L_E58:_HC_K57, and _L_H69:_HC_S106.

#### PD-L1/Avelumab (PDB ID: 5GRJ)

In PD-L1/Avelumab complex, the FMO results detected 33 PPI, which are summarized in Supplementary Table S8. The FMO results were in agreement with the previously reported 8 PPI of Liu *et al*.^30^_L_E58:_HC_Y52, _L_E58:_HC_V104, _L_E60:_LC_Y34, _L_D61:_HC_V104, _L_D61:_HC_T105, _L_D61:_HC_T106, _L_Y56:_HC_G55 and _L_N63:_HC_T103.

#### PD-L1/KN035 complex (PDB ID: 5JDS)

In PD-L1/KN035 complex, the FMO results detected 25 PPI, which are summarized in Supplementary Table S9, and 17 water-bridge interactions, which are summarized in Supplementary Table S10.

The FMO results were in agreement with the published site-directed mutagenesis studies^31,32^. The FMO results showed 21 PPI related with the mutagenesis data, where the mutations on _L_Y56, _L_E58, _L_D61, _L_N63, _L_Q66, _L_R113, _L_M115, _L_Y123, and _L_R125 in PD-L1 made the lower binding affinity to KN035. In particular, it was reported that I54A in PD-L1 decreased binding affinity between PD-L1 and KN035. The FMO results showed 4 intra-protein interactions with _L_I54 in PD-L1: _L_I54:_L_L50, _L_I54:_L_H69, _L_I54:_L_G70, and _L_I54:_L_Y118. Because _L_I54 in β-sheet 4 may stabilize the structure of PD-L1 by interacting with _L_L50 in loop, _L_H69 next to β-sheet 5, _L_G70 next to β-sheet 6, and _L_Y118 next to β-sheet 3, I54A mutation may make β-sheet flexible and the stability unstable.

#### PD-L1/Atezolizumab (PDB ID: 5XXY)

In PD-L1/Atezolizumab complex, the FMO results detected 40 PPI, which are summarized in Supplementary Table S11. The FMO results were in agreement with the previously reported 12 PPI of Zhang *et al*.:^32^_L_A52:_LC_Y93, _L_Y56:_HC_W50, _L_E58:_HC_Y54, _L_E58:_HC_G55, _L_E58:_HC_S57, _L_D61:_HC_G55, _L_N63:_HC_S57, _L_Q66:_HC_S57, _L_Y123:_HC_W101, _L_R125:_HC_S30, _L_R125:_HC_D31, and _L_R125:_HC_Y54.

The FMO results were in agreement with with the sited-directed mutagenesis studies^32^. The FMO results showed 19 PPI related with the mutagenesis data, where the mutations of I54A, Y56A, E58A, N63A, Q66A, R113A, M115A, Y123A, and R125A in PD-L1 reduced the binding affinity between PD-L1 and atezolizumab. The mutations except I54A and M115A were correlated well with the FMO results. The FMO results showed three and four intra-protein interactions with I54 and M115 in PD-L1, respectively: three non-PPI in _L_I54: _L_I54:_L_L50, _L_I54:_L_V68, and _L_I54:_L_Y118: and four non-PPI in _L_M115: _L_M115: _L_W57, _L_M115: _L_R113, _L_M115: _L_Y123, and _L_M115: _L_K124. Because _L_I54 stabilizes PD-L1 by interacting with the end of β-sheets (_L_V68 and _L_Y118) and loop (_L_L50), I54A mutation may make PD-L1 unstable. Because _L_M115 stabilizes the hot spot regions of PD-L1, M115A mutation may make the hot regions of PD-L1 flexible.

#### PD-L1/Durvalumab (PDB ID: 5X8M)

In PD-L1/Durvalumab complex, the FMO results detected 26 PPI, which are summarized in Supplementary Table S12, and 11 water-bridge interactions, which are summarized in Supplementary Table S13. The FMO results were in agreement with the previously reported 8 PPI of Lee *et al*.^33^_L_D26:_LC_R28, _L_E58:_HC_K52, _L_V111:_HC_E105, _L_R113:_HC_E57, _L_K124:_LC_S94, _L_K124:_LC_W97, _L_R125:_HC_G104, and _L_R125:_LC_G93.

#### The common hot spot region in PD-L1 against five PD-L1-antibodies and PD-1

In order to investigate the common hot spot regions among five PD-L1-antibodies and PD-1, we illustrated the FMO results in Fig. 3. The five PD-L1-antibodies have PPI with _L_Y56 and _L_E58 in common, while four of them had PPI with _L_D61, _L_Q66, _L_R113, _L_Y123, and _L_R125 in PD-L1. In the wild-type complex, _L_D61 had no PPI between PD-1 and PD-L1, but had intra-protein interactions with _L_R113 (−14.636 kcal/mol), which may contribute to the stability in the first hot spot in PD-L1. Some antibodies had PPI with _L_E60, _L_H69, and _L_D122. The FMO results showed that most PPI between PD-L1-antibodies and PD-L1 focused on the PPI on the first hot spot between PD-1 and PD-L1.

### Hot spot region which small ligand inhibitors occupied in PD-L1

In order to narrow down hot spot regions in PD-1/PD-L1 complex, we performed FMO calculations of two peptide complexes in complex with PD-L1 and four small-molecule complexes in complex with two PD-L1 proteins, and analyzed them with 3D-SPIEs method in case of peptide inhibitors and traditional bar graph method in case of small-molecule inhibitors.

#### PD-L1/peptide-57

In PD-L1/peptide-57 complex, the FMO results detected 9 strong interactions between peptide-57 and PD-L1 with 9 residues and 6 water-bridge interactions: 9 residues: _L_Y56, _L_E58, _L_N63, _L_Q66, _L_E71, _L_E72, _L_D73, _L_R113, and _L_M115 are summarized in Supplementary Table S14: and 6 water-bridge interactions are summarized in Supplementary Table S15. The FMO results were in agreement in with the published mutagenesis data, where peptides which lack W8 and W10 residues had relatively low binding affinity^34^. _57_W8:_L_Y56, _57_W8:_L_Q66, _57_W10:_L_R113 and _57_W10:_L_M115.

#### PD-L1/peptide-71

In PD-L1/peptide-71 complex, the FMO results detected 10 strong interactions between peptide-71 and PD-L1 with 10 residues and 4 water-bridge interactions: 10 residues: _L_V55, _L_Y56, _L_E58, _L_D61, _L_K62, _L_N63, _L_Q66, _L_R113, _L_M115, and _L_Y123 are summarized in Supplementary Table S16: and 4 water-bridge interactions are summarized in Supplementary Table S17. The FMO results were in agreement in with the published mutagenesis data: _71_Y11:_L_E58, _71_Y11:_L_R113, _71_F1:_L_Y56, _71_F1:_L_E58, _71_F1:_L_Q66, _71_NMePhe7(MEA7):_L_V55, MEA7:_L_M115, MEA2:_L_Q6, and _71_NMeNle3(9KK3):_L_Q6. In the mutagenesis data, the exchange of the central _71_Y11 to alanine, lack of _71_F1 and _71_NMePhe7, and methylation on _71_NMePhe2 and _71_NMeNle3 caused the lower binding affinity^34^.

#### Four small molecule inhibitors in complex with two PD-L1 proteins

There were the reported four nonpeptidic small-molecule inhibitors in complex with two PD-L1 proteins by Bristol-Myers-Squibb (BMS-8, BMS-37, BMS-200, and BMS-202). Because it was reported that these compounds made dimerization of two PD-L1 proteins, we used superscripts ‘A’ and ‘B’ to annotate protein molecules depending on the chain arrangement in the crystal structures.

**Figure 4:**
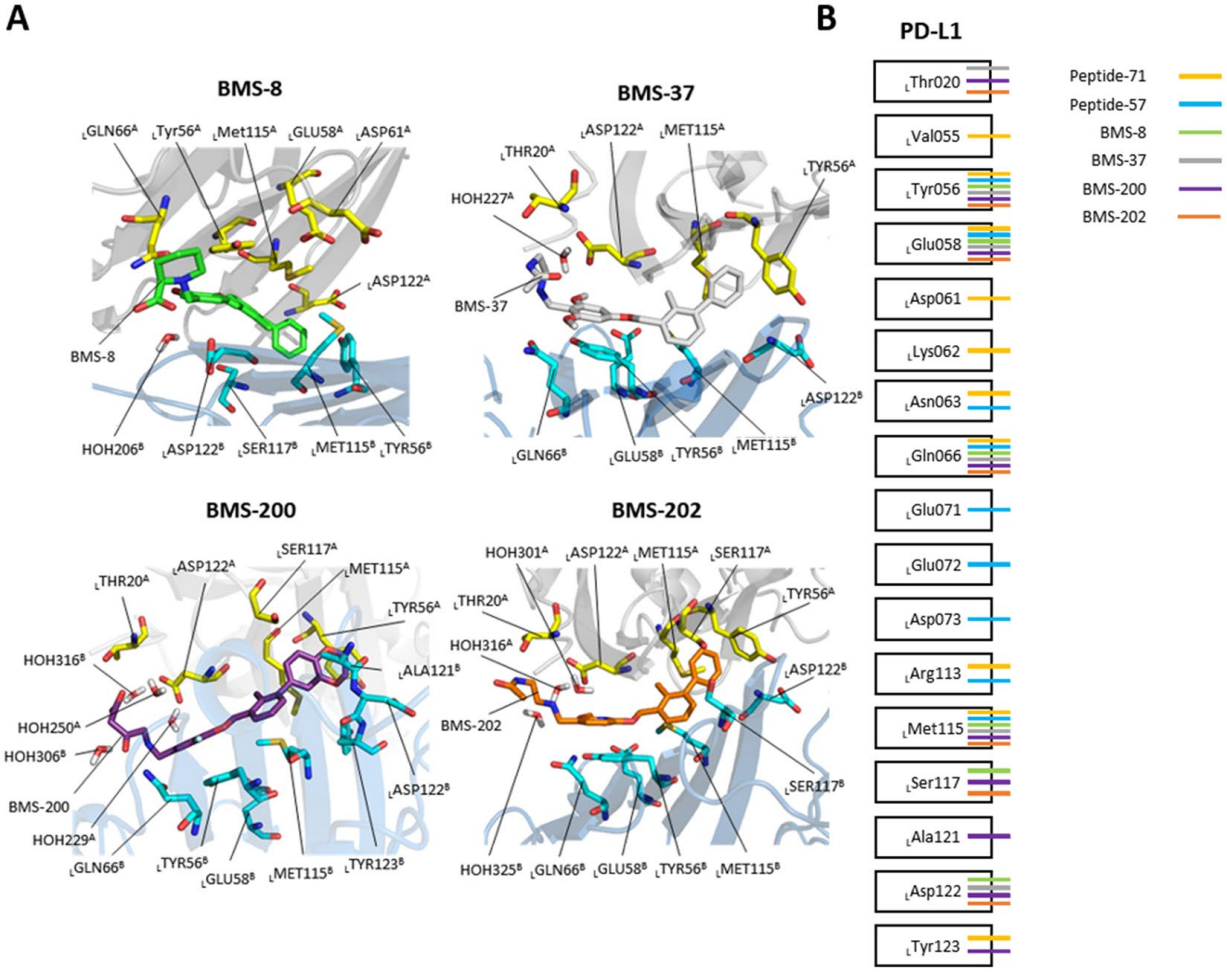
Interaction map in PD-L1, peptide inhibitors, and small-molecule inhibitors. (**A**) FMO results for BMS-8, BMS-37, BMS-200, and BMS-202. All residues attractively significant for the PIE values calculated by FMO are highlighted as sticks within 5 Å from each ligand. Model A and B for PD-L1 dimer are shown in gray and blue ribbon, respectively. The carbon atoms of BMS-8, BMS-37, BMS-200, and BMS-202 are colored in green, gray, violet, and orange, while the carbon atoms of model A and B of PD-L1 are in yellow and cyan. Nitrogen and oxygen atoms are shown in blue and red. Hydrogen atoms are omitted except water molecules. All interactions shown here have attractive PIE value more stable than −3.0 kcal/mol, whose magnitudes are ignored. (**B**) Interactions of PD-L1 with peptide inhibitors and small-molecule inhibitors are shown, where all interactions shown in this map have attractive PIE value more stable than −3.0 kcal/mol, whose magnitudes are ignored.

The FMO results of BMS compounds and two PD-L1 proteins are illustrated in Fig. 4, and their interactions are organized in Supplementary Table S18–S21. In BMS-8/PD-L1 complex, the FMO results detected 10 strong interactions with PD-L1 with 9 residues and one water molecule: 9 residues: _L_Y56^A^, _L_E58^A^, _L_Q66^A^, _L_M115^A^, _L_D122^A^, _L_Y56^B^, _L_M115^B^, _L_S117^B^, and _L_D122^B^: and one water molecule: HOH206. In BMS-37/PD-L1 complex, the FMO results detected 10 strong interactions with PD-L1 with 9 residues and one water molecule: 9 residues: _L_T20^A^, _L_Y56^A^, _L_M115^A^, _L_D122^A^, _L_Y56^B^, _L_E58^B^, _L_Q66^B^, _L_M115^B^, and _L_D122^B^: and one water molecule: HOH227. In BMS-200/PD-L1 complex, the FMO results detected 16 strong interactions with PD-L1 with 12 residues and four water molecules: 12 residues: _L_T20^A^, _L_Y56^A^, _L_M115^A^, _L_S117^A^, _L_D122^A^, _L_Y56^B^, _L_E58^B^, _L_Q66^B^, _L_M115^B^, _L_A121^B^, _L_D122^B^, and _L_Y123^B^: and four water molecules: HOH229, HOH250, HOH306, and HOH316. In BMS-202/PD-L1 complex, the FMO results detected 13 strong interactions with PD-L1 with 10 residues and three water molecule: 10 residues: _L_T20^A^, _L_Y56^A^, _L_M115^A^, _L_S117^A^, _L_D122^A^, _L_Y56^B^, _L_E58^B^, _L_Q66^B^, _L_D73^B^, _L_M115^B^, and _L_D122^B^: and three water molecules: HOH301, HOH316, and HOH325.

### Hot spot region in PD-1/PD-L1 integrated with 3D-SPIEs-based interaction map

To integrate information between PPI and small-molecule-protein interactions, we made 3D-SPIEs-based interaction map. (see Figs 3 and 4). In the wild-type complex, we found that there were two hot spot regions. In the first hot spot, most of PD-L1-antibodies, peptides, and small-molecule inhibitors have common significant interactions with _L_Y56, _L_E58, and _L_Q66. In the second hot spot, most of PD-L1-antibodies had interactions with _L_D122 and _L_R125, while most of peptides and small-molecule inhibitors had interactions with _L_D122. According to the map, the first hot spot might be the most important hot spot on PPI interface in the PD-1/PD-L1 complex. Notably, _L_M115, located between the first and the second hot spot regions on PPI interface, had significant interactions with all of peptides and small-molecule inhibitors, while there was no PPI in wild-type complex, while two of the five PD-L1-antibodies had PPI with _L_M115. In intra-protein interactions in PD-L1 of the wild-type, _L_M115 interacted with _L_W57 (−17.157 kcal/mol), _L_Y123 (−7.154 kcal/mol), and _L_K124 (−16.566 kcal/mol). It indicated that _L_M115 may be also important for maintenance of the first hot spot region in PD-L1 and play a role of pivot, connecting the first and the second hot spots, for small ligands.

## Discussion

Given the successful prospects in clinical studies of monoclonal antibodies targeting the PD-1/PD-L1 axis, several agents to block the PD-1/PD-L1 axis have been developed. The understanding of PPI between PD-1 and PD-L1 is vital for successful structure-based drug design. Even though the crystal structures of PD-1/PD-L1 complex were solved, it has been still a challenge to find small-molecule inhibitors. Visual inspection or traditional molecular mechanic approaches do not always give a clear answer, especially in protein-protein interactions. In the present study, we made the 3D-SPIEs-based interaction map in PD-1/PD-L1, which integrated PPI and small-molecule-protein interactions at a quantum-mechanical level. In the map, it indicated that the first hot spot region is important for inhibiting PPI in PD-1/PD-L1, which all antibodies and small-ligands focused on. The integrated information can be used in the design of new compounds in hit-to-lead or lead optimization stages for targeting PD-1/PD-L1 in drug discovery.

Water molecules play a crucial role in the protein-protein interactions, though they can be quickly exchanged with bulk solvent^35^. Here, we analyzed water molecules which were resolved in the crystal structures. Using the FMO method to analyze the water-bridge interactions in protein-protein system can allow us to identify water molecules which are energetically favorable or unfavorable. And this information can be used to design several agents that can interact with certain water molecules or displace the interactions. However, identification and verification of water molecules depends on the resolution of X-ray crystal structures. The FMO method is suitable for analyzing the roles of already predicted, or identified, water molecules which have been determined by other computational methods^15^.

FMO/PIEDA method has been a practical tool for rational structure-based drug design, because it offers a precise and comprehensive information on the protein-ligand interactions^15,19^. The information from the FMO/PIEDA method allows us to distinguish whether the interactions are strong, moderate, weak or not, and whether they are attractive or repulsive between the ligand and its neighboring residues. Furthermore, it can be used as an efficient *ab initio* tool for design, assessment and filtering of new compounds, which may reduce the effort and the cost of chemical synthesis in drug discovery.

Even though the FMO method was successfully applied to the protein-protein interaction of influenza hemagglutinin^19,24,36^, it has not been generally employed in other PPI cases. Although the analysis scheme, summing all PIEs over all residues of a partner protein, facilitated the analysis of PPI with a bar graph, it required the huge computational cost. Here, we introduced the 3D scattered pair interaction energies (3D-SPIEs) to sort and analyze PIEs between fragments, which provided two advantages. Firstly, it allowed the FMO method to be applied easily to protein-protein systems due to lower computational cost from a-fragment-per-a-residue scheme. It would provide a protocol to analyze PPI, which can be applied to protein-RNA and protein-DNA complexes in future. Secondly, most protein-protein interactions, detected by the FMO/3D-SPIEs, method were in agreement with the published reports and mutagenesis data. Not only providing the reported PPI, it had additional advantage of distinguishing inter-protein (PPI) and intra-protein (non-PPI) interactions. In some cases, the alanine scanning experiments could be inconclusive, because the mutations could affect the binding free energy by a mechanism unrelated with PPI^37^. Some mutations might destabilize the unbound state of protein and alter its conformation, so hot spots identified by alanine scanning experiments could be false positives^37^. It indicated that the application of FMO method to PPI can predict site-directed mutagenesis study results, and furthermore provide intra-protein interactions affecting stability. It would provide the starting points to modify proteins and nucleic-acids as a powerful tool in protein-engineering and nucleic-acid-engineering for the higher binding affinity.

In summary, the outcome of this study provided three points. Firstly, as a protocol, the FMO/3D-SPIEs method can be applied to analyze hot spot region of PPI. Secondly, in analyzing case-by-case PPI, the FMO/3D-SPIEs results had a qualitative correlation with site-directed mutagenesis results, and additionally provided intra-protein interactions for stability. Thirdly, the integrated information with PPI and small-ligand interactions in PD-1/PD-L1, the FMO/3D-SPIEs results provided valuable information for hot spot regions in PD-1/PD-L1, and designing new potential small-molecule targeting PD-1/PD-L1. Therefore, the FMO/3D-SPIEs method allowed the FMO method to be used to understand not only the nature of protein-ligand complexes, but also protein-protein complexes. The FMO method can be hereby used to analyze hot spots in PPI, protein-RNA, and protein-DNA, and provide new direction to hit-to-lead, lead optimization, protein engineering, and nucleic-acid engineering in future.

## Computational Methods

### Structure preparation

X-ray crystal structures were collected from the Protein Data Bank (PDB), a wild-type PD-1/PD-L1 complex (PDB ID: 4ZQK): two crystal structures of PD-1/mAb complexes: nivolumab (PDB ID: 5WT9) and pembrolizumab (PDB ID: 5B8C), five crystal structures of PD-L1/mAb complexes: BMS-936559 (PDB ID: 5GGT), avelumab (PDB ID: 5GRJ), KN035 (PDB ID: 5JDS), atezolizumab (PDB ID: 5XXY), and durvalumab (PDB ID: 5X8M), two crystal structures of the PD-L1/peptide complexes: peptide-71 (PDB ID: 5O45) and peptide-57 (PDB ID: 5O4Y), and four crystal structures of small molecule inhibitor in complex with two PD-L1 molecules: BMS-8 (PDB ID: 5J8O), BMS-37 (PDB ID: 5N2D), BMS-200 (PDB ID: 5N2F), and BMS-202 (PDB ID: 5J89). All the missing side chains and loops of the crystal structures were filled using Prime implemented in *Maestro* program^38,39^. Hydrogen atoms were added to the crystal structures at physiological pH (7.4) and their positions were optimized with the PROPKA implemented in Maestro program^40^. Then the restrained energy minimization was performed on PD-1/mAb and PD-L1/mAb crystal structures with OPLS3 in *Maestro* program with 0.3 Å RMSD (root mean square deviation)^41^. Water molecules in the crystal structures were included in FMO calculations to explore their roles in protein-protein interactions.

### FMO calculations

All FMO calculations were performed with the version Aug 18, 2016 in GAMESS^42^. Two-body FMO method was applied to PD-1/PD-L1, PD-1/mAb, PD-L1/mAb, and PD-L1/small-ligand complexes to investigate key interactions at the second order Møller-Plesset perturbation (MP2)^43^ and polarizable continuum model (PCM)^44^ with 6-31G** basis set (FMO-MP2/6-31G**/PCM level). The implicit solvation model (PCM) was employed with explicitly expressed water molecules present in the X-ray crystal structures. In FMO calculations of PD-1/mAb and PD-L1/mAb, only Fv domains of mAb and crystallographic water molecules around the region were included. All inputs files were prepared with an in-house code in compliance with the hybrid orbital projection (HOP)^45^ scheme fragmentation. Each residue and water molecule was defined as a fragment and two cysteine residues forming the disulfide bridge were defined as a fragment (a-fragment-per-a-residue fragmentation scheme).

Two body FMO calculation consists of four sequential steps on (i) fragmentation, (ii) fragment (monomer) self-consistent field (SCF) calculation, (iii) fragment pair (dimer) SCF calculation, and iv) total property, including energy and gradient, evaluation^15^.

(i) Fragmentation is a starting point for FMO method. In a-fragment-per-a-residue fragmentation scheme, each residue of protein, ligand and water molecule can be defined as a fragment except two cysteine residues forming disulfide bond. It should be noted that each fragment, representing amino acid residues, is different from the normal assignment for the amino acid residues^45,46^. In FMO calculations, all the residues in protein are divided at the *sp3* bond of Cα position, not peptide bond, based on the hybrid orbital projection (HOP)^45^ scheme, where the fragmentation is performed at an atom, not midway of bond^47^ in Fig. 1. If the main-chain carbonyl oxygen contributes to hydrogen bond, the interaction will be shifted to the next amino acid by HOP fragmentation, because the carbonyl group of the amino acid belongs to the next amino acid in the scheme. The scheme was introduced for reducing computational cost significantly and correcting errors from projection operator^19,45–47^.
(ii) All the MOs on each fragment (monomer) are optimized by self-consistent-field (SCF)^48^ theory in the whole electrostatic field and all the electron densities are self-consistently solved through self-consistent-charge (SCC) iterations^17,24^.
(iii) Schrödinger equations for fragment pair (dimer) are self-consistently solved in the same way as for the second step, independently^24^. Dimer fragments are made of two monomer fragments. The difference between the third step (dimer) and the second step (monomer) is that the Hamiltonian operator of dimer includes the electrostatic potential generated by surrounding *N-2* monomers and that of monomer includes it generated by surrounding *N-1* monomers, where *N* means the number of fragments.
(iv) In total property evaluation step, all MO calculations for monomer and dimer are pieced together to generate whole picture of the system. FMO provides the pair interaction energies (PIEs) between fragments. In pair interaction energy decomposition analysis (PIEDA), PIE or $\Delta {E}_{ij}^{int}$, between fragments *i* and *j*, indicates a sum of four energies including the electrostatic $(\varDelta {E}_{ij}^{es})$, exchange-repulsion $(\varDelta {E}_{ij}^{ex})$, charge transfer $(\Delta {E}_{ij}^{ct+mix})$, and dispersion $(\varDelta {E}_{ij}^{di})$ term, which has the same contributions as in EDA^18^. Solvation energy $(\varDelta {G}_{sol})$ can be added to PIE, which is shown in Eq. (1).

1 $$\mathrm{PIE}\,(\Delta {E}_{ij}^{int})=\Delta {E}_{ij}^{es}+\Delta {E}_{ij}^{ex}+\Delta {E}_{ij}^{ct+mix}+\Delta {E}_{ij}^{di}+\Delta {G}_{sol}$$

### 3D Scattered pair interaction energies (3D-SPIEs)

The main advantage of FMO is that it can account for the energy and structural fitness that stabilizes the complex by calculating the interaction energy (PIE) between the fragments, which make up the protein-protein or protein-ligand complexes. Therefore, it is necessary to sort and analyze the information obtained from the FMO calculations in an easy-to-understand way. The FMO results consist of three-dimensional data, whose elements are pair interaction energies and names of two fragments. In traditional and powerful way, the FMO results can be analyzed with two-dimensional bar graphs^23,36^, because name of ligand fragment can be omitted in protein-ligand system. However, names of residue fragments in PPI cannot be omitted because they include crucial information. For facilitating analysis of PPI and representing the results with two-dimensional bar graphs, a partner protein can be defined as a fragment, which requires huge computational cost. In order to reduce computational cost and obtain interaction information of all residue pairs in PPI system, we used a-fragment-per-a-residue fragmentation scheme and devised 3-dimensional scattered pair interaction energies (3D-SPIEs) analysis method. (see Fig. 1) In 3D-SPIEs, we sorted out PIE with distance between fragments and energy value information to find important PPI information from all fragment interaction information. We calculated the distance between fragments with single-linkage clustering, where the distance between two fragments equals to the distance between the two atoms that are nearest apart from each other. To make 3D-SPIEs, we used 3D scatter plot graph implemented in plotly^49^. In plots from 3D-SPIEs, the interactions are marked as dots which have 5 components: x, y, z, energy values, and color. The x and y axes indicate name of fragments (e.g. the residues’ name of PD-1 and PD-L1), z axis indicates the distance between two fragments, and the dots have color according to their PIEs values. And then, PIEDA information is shown when dots are hovered.

## Supplementary information

Supporting Information

## Data Availability

All 3D-SPIEs results and python code in this work are available in GitHub (https://github.com/hclim0213/3D-SPIEs).
