# Supplementary material for: Investigation of protein-protein interactions and hot spot region between PD-1 and PD-L1 by fragment molecular orbital method
Source: Sci Rep. 2019 Nov 13;9:16727. doi: 10.1038/s41598-019-53216-z (PMC6853875; doi:10.1038/s41598-019-53216-z)
Supplement: Supplementary file 1 — Supporting Information [file 41598_2019_53216_MOESM1_ESM.pdf]

Supporting Information for

Investigation of protein-protein interactions and hot spot region between PD-1 and PD-L1 by fragment molecular orbital method

Hocheol Lim[1], Jungho Chun[1], Xuemei Jin[2], Jongwan Kim[1, 2], JeongHyeok Yoon[3], and Kyoung Tai No[1, 2, ‡]

[1]Department of Biotechnology, Yonsei University, Seoul, Republic of Korea

[2]Bioinformatics and Molecular Design Research Center (BMDRC), Yonsei University, Seoul, Republic of Korea

[3]Pharos I&BT Co., Ltd., Anyang-si, Gyeonggi-do, 14059, Korea

[‡]Corresponding Author.

E-mail: ktno@bmdrc.org (K. T. No)

The supporting information for 'investigation of protein-protein interactions and hot spot region between PD-1 and PD-L1 by fragment molecular orbital method' includes Table S1 – S21 for PIEDA results and water-bridge interactions among PD-1, PD-L1, and their inhibitors.

In this study, there are many abbreviations as follows. 3D-SPIEs, 3-Dimensional Scattered Pair Interaction Energies; EDA, Energy Decomposition Analysis; FMO, Fragment Molecular Orbitals method; HOP, Hybrid Orbital Projection; mAb, monoclonal Antibody; MM, Molecular Mechanics; PCM, Polarizable Continuum Model; PD-1, Programmed cell Death protein 1; PDB, Protein Data Bank; PD-L1, programmed Death Ligand-1; PIE, Pair Interaction Energy; PIEDA, Pair Interaction Energy Decomposition Analysis; PPI, Protein Protein Interactions; QM, Quantum Mechanics; RMSD, Root Mean Square Deviation of atomic positions; SCC, Self-Consistent-Charge; SCF, Self-Consistent Field.

Table S1 The PIEDA of wild-type PD-1/PD-L1 complex

PD-1	PD-L1	PIE	ΔE^{es}	ΔE^{ex}	ΔE^{ct+mix}	ΔE^{di}	ΔG^{sol}
Val064	Asp122	-3.946	-2.152	0.001	-0.075	-0.136	-1.585
Asn066	Asp122[b]	-18.440	-19.657	6.758	-2.644	-3.121	0.224
Tyr068	Asp122[a]	-24.045	-25.977	13.453	-5.636	-5.735	-0.151
Met070	Arg125	-7.473	-5.874	1.531	-0.520	-1.886	-0.725
Ser073	Asp026	-17.694	-13.337	0.515	-1.996	-1.591	-1.284
Asn074	Arg125	-10.826	-3.526	0.112	-0.356	-0.679	-6.377
Gln075	Asp026[a]	-23.404	-23.405	4.601	-3.459	-3.643	2.502
	Lys124	-6.318	-2.800	2.052	-1.809	-3.603	-0.158
	Arg125[b]	-31.290	-34.446	10.113	-4.795	-5.994	3.833
	Ile126[b]	-12.949	-14.959	6.885	-1.903	-2.806	-0.165
Thr076	Asp122	-8.271	-8.125	-0.001	-0.021	-0.062	-0.061
Asp077	Lys124	-88.176	-97.849	5.380	-3.645	-5.272	13.210
Lys078	Thr020[b]	-37.320	-40.093	8.110	-3.343	-4.765	2.771
	Asp122	-97.338	-97.834	4.639	-3.232	-5.206	4.294
Glu084	Ala018	-34.412	-62.832	0.131	-0.874	-0.631	29.794
	Phe019	-22.275	-18.097	1.106	-2.953	-2.741	0.411
	Gly120	-8.334	1.061	0.541	-1.346	-1.586	-7.004
	Ala121	-8.604	-9.663	0.000	0.049	-0.195	1.205
Lys131	Asp073	-5.767	-15.739	-0.001	-0.076	-0.163	10.211
Ala132	Gln066[a]	-9.079	-10.216	7.773	-2.260	-4.687	0.311
Gln133	Gln066	-4.044	-3.514	0.657	-0.285	-1.000	0.098
Ile134	Tyr056[a]	-3.186	-1.756	0.650	-0.339	-1.868	0.127
	Glu058[a]	-13.224	-11.334	0.880	-1.934	-2.120	1.284
	Tyr123[a]	-3.389	0.171	2.562	-1.437	-4.753	0.068
Lys135	Glu058	-21.908	-25.791	-0.001	-0.009	-0.114	4.006
Glu136	Arg113[a]	-56.967	-67.696	2.256	-1.826	-2.903	13.202
	Tyr123[a]	-29.400	-29.698	10.736	-4.650	-5.800	0.012
	Arg125[a]	-88.219	-96.646	7.258	-5.329	-4.636	11.134

All energies are in kcal/mol.

The calculation has MP2/6-31G**/PCM level

[a] The interactions were reported in ref 7

[b] The interactions were shifted by HOP fragmentation

Table S2 Water-bridge interaction in PD-1/PD-L1 complex

PIE	PD-1	Water molecules	PD-L1	PIE
-9.941	Ile134[a]	HOH202	Tyr056[a]	-7.769
			Glu058[a]	-18.794
			Asp061	-3.257
-14.333	Asn066[a]	HOH203	Ala121[a]	-7.517
-12.574	Lys078			

All energies are in kcal/mol.

The calculation has MP2/6-31G**/PCM level

[a] The interactions were reported in ref 7

Table S3 The PIEDA of PD-1/Nivolumab complex

PD-1	Nivolumab	PIE	ΔE^{es}	ΔE^{ex}	ΔE^{ct+mix}	ΔE^{di}	ΔG^{sol}
Leu025	$_{HC}$Trp052[a]	-8.020	-5.679	1.363	-0.883	-2.159	-0.662
	$_{HC}$Lys057[a]	-12.298	-5.238	5.461	-2.012	-2.058	-8.450
Asp026	$_{HC}$Lys057	-33.594	-49.658	0.130	-1.301	-1.001	18.237
	$_{LC}$Trp094	-4.839	-7.077	-0.001	0.062	-0.159	2.336
Ser027	$_{LC}$Arg096	-9.336	-8.955	-0.001	0.030	-0.067	-0.343
Pro028	$_{HC}$Trp052	-8.811	-5.361	8.913	-3.867	-9.023	0.527
Asp029	$_{HC}$Asn031	-4.968	-3.730	-0.001	-0.009	-0.059	-1.168
	$_{HC}$Gly033[a]	-24.853	-28.605	12.141	-4.577	-5.190	1.379
	$_{HC}$Tyr053	-15.492	-14.831	7.436	-3.571	-5.447	0.922
	$_{HC}$Asn099	-44.153	-42.391	7.548	-5.007	-5.457	1.154
Arg030	$_{HC}$Ser030[a]	-5.587	-1.590	0.173	-0.587	-0.917	-2.666
	$_{HC}$Asn031[a]	-39.769	-42.963	9.945	-5.807	-8.346	7.402
	$_{HC}$Ser032[b]	-6.425	-8.273	6.973	-0.876	-3.320	-0.929
	$_{HC}$Asp054	-33.271	-53.493	-0.001	-0.030	-0.431	20.685
Pro031	$_{HC}$Asn031	-3.503	-3.511	0.000	0.003	-0.191	0.196
Ser060	$_{HC}$Asn031	-5.349	-3.133	1.789	-1.519	-1.952	-0.534
Ala129	$_{HC}$Asp101	-3.039	-2.558	0.149	-0.570	-0.518	0.458
Pro130	$_{HC}$Asp100	-4.510	-4.245	0.001	-0.066	-0.351	0.152
	$_{HC}$Asp101	-6.093	-3.715	1.661	-1.504	-2.782	0.246
	$_{HC}$Tyr102	-6.895	-4.130	4.504	-0.826	-6.019	-0.424
	$_{LC}$Thr056[b]	-4.405	-2.170	2.833	-2.092	-3.108	0.133
Lys131	$_{HC}$Asp100[a]	-47.739	-59.225	6.492	-3.205	-4.100	12.299
	$_{HC}$Asp101[a]	-94.442	-108.606	9.127	-3.741	-7.583	16.361
	$_{LC}$Tyr049[a]	-14.049	-11.087	6.225	-2.350	-6.844	0.006
	$_{LC}$Asp050	-36.277	-50.776	0.013	-0.390	-0.431	15.306
	$_{LC}$Thr056[b]	-11.119	-10.780	3.131	0.447	-3.028	-0.889
Ala132	$_{HC}$Asp101	-6.638	-4.749	0.006	0.046	-0.201	-1.740
	$_{LC}$Tyr049[a]	-3.813	-1.581	0.961	-0.807	-2.551	0.166
Gln133	$_{LC}$Tyr049	-9.668	-10.095	3.855	-1.468	-2.894	0.934

All energies are in kcal/mol.

The calculation has MP2/6-31G**/PCM level

The subscripts HC and LC indicate the heavy and light chain, respectively

[a] The interactions were reported in ref 26

[b] The interactions were shifted by HOP fragmentation

Table S4 Water-bridge interaction in PD-1/Nivolumab

PIE	PD-1	Water molecules	Nivolumab	PIE
-3.505	Asp029	^{G}HOH404	$_{HC}$Ser032	-9.776
-10.524	Pro031			
-20.791	Asp029	^{G}HOH405	$_{HC}$Ser032	-5.404
			$_{HC}$Thr098	-3.455
			$_{HC}$Asp100	-12.94
-6.703	Asp026	^{G}HOH409	$_{LC}$Trp094	-9.927
-11.111	Ser027			
-4.07	Ala129	^{H}HOH343	$_{HC}$Tyr102	-9.146
-6.864	Asp026	^{H}HOH364	$_{HC}$Lys057	-4.225
			$_{HC}$Tyr059	-10.276
-6.018	Lys131	^{L}HOH336	$_{LC}$Leu033	-5.417
			$_{LC}$Gln089	-4.301
			$_{LC}$Gln090	-11.526
			$_{LC}$Ser091	-7.029
-4.351	Lys131	^{L}HOH350	$_{LC}$Asp050	-22.135
			$_{LC}$Ser091	-8.936
-7.223	Asp026	^{L}HOH352	$_{LC}$Ser092	-10.764
-11.641	Lys131	^{L}HOH357	$_{LC}$Asp050	-25.384
-12.873	Leu128	^{L}HOH370	$_{LC}$Thr056	-7.966

All energies are in kcal/mol.

The calculation has MP2/6-31G**/PCM level

The subscripts HC and LC indicate the heavy and light chain, respectively

The superscripts G, H and L indicate the chain arrangement

Table S5 The PIEDA of PD-1/Pembrolizumab complex

PD-1	Pembrolizumab	PIE	ΔE^{es}	ΔE^{ex}	ΔE^{ct+mix}	ΔE^{di}	ΔG^{sol}
Glu061	$_{LC}$Tyr034[b]	-12.280	-3.189	5.118	-2.258	-5.767	-6.184
	$_{LC}$Tyr057[a]	-3.582	0.274	0.000	-0.202	-0.309	-3.345
Ser062	$_{LC}$Tyr057[b]	-15.439	-13.379	4.517	-3.056	-3.452	-0.070
Val064	$_{LC}$Tyr034	-18.903	-18.956	9.389	-3.663	-5.057	-0.616
	$_{HC}$Phe103[c]	-3.806	-1.993	5.227	-1.365	-5.559	-0.116
Asn066	$_{HC}$Arg102[a]	-8.779	-7.464	2.506	-1.904	-2.582	0.665
	$_{HC}$Phe103[c]	-7.510	-10.298	6.343	-0.409	-3.260	0.114
Tyr068	$_{HC}$Arg102[c]	-9.225	-7.600	3.285	-1.848	-3.427	0.365
Gln075	$_{HC}$Thr030[a]	-12.646	-12.391	4.437	-2.416	-3.050	0.775
Thr076	$_{HC}$Tyr101[a]	-6.315	-5.573	2.505	-1.287	-2.012	0.052
Asp077	$_{HC}$Asn052	-7.264	-5.571	0.040	-0.725	-0.591	-0.418
	$_{HC}$Ser054[a]	-20.758	-23.572	7.585	-4.047	-3.628	2.904
	$_{HC}$Asn055	-11.547	-6.649	0.029	-0.676	-0.770	-3.480
Lys078	$_{HC}$Tyr033[a]	-15.002	-10.741	2.633	-1.390	-3.144	-2.361
	$_{HC}$Tyr101[a]	-6.560	-3.266	1.603	-1.026	-2.987	-0.884
	$_{HC}$Arg102[c]	-9.110	-2.897	7.360	-3.127	-4.348	-6.097
	$_{HC}$Phe103[c]	-15.373	-13.580	1.157	-0.899	-1.986	-0.064
Pro083	$_{LC}$Tyr034	-4.164	-1.895	4.879	-1.664	-5.300	-0.183
Asp085	$_{HC}$Arg099[a]	-121.969	-129.140	20.531	-6.981	-7.377	0.998
	$_{HC}$Arg102[c]	-11.489	-16.723	0.000	-0.046	-0.089	5.369
Arg086	$_{LC}$Asp097[a]	-68.921	-87.219	7.265	-3.558	-5.923	20.513
Gln088	$_{HC}$Tyr033[c]	-5.131	-1.884	2.199	-1.541	-3.747	-0.158
	$_{HC}$Tyr035[b]	-16.078	-18.297	9.712	-3.588	-3.828	-0.077
	$_{HC}$Asn059[a]	-7.206	-7.013	3.460	-1.485	-2.243	0.075
	$_{HC}$Arg099	-8.688	-7.583	0.045	-0.274	-0.662	-0.215
Pro089	$_{HC}$Tyr035[c]	-4.752	-2.261	1.859	-1.369	-3.041	0.060
	$_{HC}$Thr058	-5.156	-4.129	2.126	-1.199	-1.967	0.014
	$_{HC}$Asn059	-4.004	-0.977	2.608	-1.461	-4.325	0.151
Gly090	$_{HC}$Asn052	-8.425	-6.647	0.617	-0.757	-1.148	-0.490
	$_{HC}$Gly057	-3.176	-3.095	1.189	-0.638	-0.509	-0.124
Gln091	$_{HC}$Asn059	-4.188	-3.845	0.045	0.043	-0.329	-0.103
Ile126	$_{HC}$Arg102[c]	-5.668	-4.222	1.543	-0.429	-2.362	-0.198
Ala129	$_{LC}$Tyr053	-17.199	-16.870	6.716	-3.238	-3.628	-0.179
Pro130	$_{LC}$Tyr057	-3.213	-1.944	1.192	-0.380	-2.445	0.365
Ala132	$_{HC}$Asp100	-4.275	-3.163	-0.001	0.048	-0.017	-1.141
Ile134	$_{HC}$Arg102[c]	-5.235	1.650	2.851	-1.021	-3.915	-4.800
Glu136	$_{HC}$Arg102	-39.192	-52.576	-0.001	-0.054	-0.132	13.572

All energies are in kcal/mol.

The calculation has MP2/6-31G**/PCM level

The subscripts HC and LC indicate the heavy and light chain, respectively

[a] The interactions were reported in ref 27

[b] The interactions were shifted by HOP fragmentation

[c] The interactions were reported in ref 28

Table S6 Water-bridge interaction in PD-1/Pembrolizumab

PIE	PD-1	Water molecules	Pembrolizumab	PIE
-20.188	Asp077	^{C}HOH404	$_{HC}$Asn055	-8.167
-8.419	Thr076	^{C}HOH405	$_{HC}$Tyr101	-5.010
-23.505	Glu136		$_{HC}$Arg102	-22.132
-8.297	Gln088	^{C}HOH408	$_{HC}$Asn052	-4.832
-10.283	Gly090		$_{HC}$Asn055	-6.960
-6.086	Gln091			
-10.442	Asp085	^{C}HOH411	$_{LC}$Arg096	-6.690
-3.945	Arg086			
-6.469	Gln099	^{C}HOH414	$_{LC}$Ser032	-7.828
-9.927	Ser087	^{C}HOH417	$_{HC}$Asn059	-7.535
-11.911	Pro089			
-7.141	Arg086	^{C}HOH419	$_{LC}$Arg096	-5.206
-3.309	Ser087			
-10.940	Ser087	^{C}HOH421	$_{LC}$Ser095	-4.282
			$_{HC}$Arg099	-4.346
			$_{HC}$Asp104	-3.174
-6.084	Lys131	^{C}HOH422	$_{LC}$Tyr053	-7.109
-8.027	Ala132			

All energies are in kcal/mol.

The calculation has MP2/6-31G**/PCM level

The subscripts HC and LC indicate the heavy and light chain, respectively

The superscript C indicates the chain arrangement

Table S7 The PIEDA of BMS-936559/PD-L1 complex

PD-L1	BMS-936559	PIE	ΔE^{es}	ΔE^{ex}	ΔE^{ct+mix}	ΔE^{di}	ΔG^{sol}
Asp049	$_{HC}$Ser104	-4.704	3.117	1.566	-1.154	-1.749	-6.483
	$_{HC}$Gly105[a]	-26.106	-27.028	8.119	-3.955	-3.698	0.456
	$_{LC}$Tyr032[a]	-26.436	-21.093	7.901	-4.019	-5.045	-4.180
Ala052	$_{HC}$Pro107	-6.266	-5.534	2.016	-0.968	-2.178	0.398
Tyr056	$_{HC}$Lys057	-6.029	-6.858	2.030	-1.324	-2.492	2.614
	$_{HC}$His059[a]	-13.155	-13.479	7.588	-2.587	-4.059	-0.619
Glu058	$_{HC}$Lys057[a]	-81.784	-98.690	0.994	-3.375	-2.919	22.205
Gln066	$_{HC}$Lys057	-11.069	-13.310	0.002	-0.008	-0.095	2.342
His069	$_{HC}$Ser106[a]	-14.659	-16.859	9.688	-3.140	-4.365	0.018
	$_{HC}$Pro107	-3.703	-2.865	0.146	-0.090	-1.023	0.129
	$_{HC}$Phe108	-3.336	-2.292	0.063	-0.246	-0.790	-0.072
	$_{LC}$Asn093	-6.134	-3.897	3.670	-2.104	-4.038	0.236
	$_{LC}$Trp094	-5.582	-1.265	4.812	-1.150	-8.011	0.032
Glu071	$_{LC}$Asn093	-8.260	-1.024	0.068	-1.252	-0.747	-5.306
Arg113	$_{HC}$Phe055	-5.104	-11.835	0.009	-0.234	-0.436	7.393
	$_{HC}$Gly056	-7.200	-8.391	0.002	-0.170	-0.162	1.521
Asp122	$_{HC}$Ile054	-3.410	-2.065	0.860	-0.746	-1.570	0.110

All energies are in kcal/mol.

The calculation has MP2/6-31G**/PCM level

The subscripts HC and LC indicate the heavy and light chain, respectively

[a] The interactions were reported in ref 29

Table S8 The PIEDA of Avelumab/PD-L1 complex

PD-L1	Avelumab	PIE	ΔE^{es}	ΔE^{ex}	ΔE^{ct+mix}	ΔE^{di}	ΔG^{sol}
Cys040	$_{HC}$Tyr052	-3.060	-2.874	0.995	-1.079	-1.488	1.386
	$_{HC}$Phe059	-5.626	-4.946	0.895	-1.080	-1.830	1.336
	$_{LC}$Ser097	-4.444	-4.083	0.460	-0.773	-1.425	1.377
	$_{LC}$Thr098	-3.048	-6.490	-0.002	-0.159	-0.132	3.735
Tyr056	$_{HC}$Tyr052	-4.647	-1.312	2.475	-1.466	-4.356	0.012
	$_{HC}$Pro053	-4.107	-2.085	1.821	-1.032	-2.974	0.163
	$_{HC}$Gly055[b]	-15.266	-18.766	9.933	-2.766	-3.370	-0.297
Glu058	$_{HC}$Tyr052[a]	-34.723	-33.867	11.382	-6.653	-5.944	0.359
	$_{HC}$Val104[a]	-8.571	-1.107	3.259	-2.294	-4.140	-4.289
Glu060	$_{HC}$Val104	-3.823	-2.277	-0.001	0.011	-0.133	-1.423
	$_{LC}$Tyr032	-9.616	1.213	1.779	-2.681	-3.502	-6.426
	$_{LC}$Tyr034[a]	-31.027	-28.601	10.477	-6.069	-6.805	-0.028
	$_{LC}$Tyr093	-4.306	-2.288	3.100	-1.607	-3.268	-0.244
Asp061	$_{HC}$Val104[a]	-15.412	-9.395	1.971	-2.001	-3.184	-2.803
	$_{HC}$Thr105[a]	-37.466	-34.641	9.561	-5.066	-6.091	-1.229
	$_{HC}$Thr106[a]	-26.084	-21.194	4.278	-2.815	-4.146	-2.207
	$_{LC}$Tyr034	-6.208	-1.811	7.496	-3.502	-7.379	-1.012
	$_{LC}$Tyr093	-9.610	-5.705	3.498	-0.945	-6.763	0.306
	$_{LC}$Arg099	-76.718	-80.484	2.443	-2.498	-2.868	6.690
Lys062	$_{HC}$Thr103	-10.186	-8.068	4.681	-3.140	-3.361	-0.298
	$_{HC}$Val104	-7.175	-11.077	6.765	-0.026	-4.404	1.568
	$_{LC}$Tyr034	-5.035	-5.901	2.312	-0.877	-3.294	2.725
Asn063	$_{HC}$Thr103[b]	-13.213	-19.890	15.182	-3.443	-4.314	-0.747
Gln066	$_{HC}$Pro053	-3.099	-2.009	1.361	-1.020	-1.951	0.519
His069	$_{HC}$Ser054	-3.824	-2.918	0.267	-1.053	-1.258	1.139
Asp073	$_{HC}$Ser031	-4.520	2.112	0.863	-1.443	-1.335	-4.716
Lys075	$_{HC}$Thr028	-5.438	-0.934	0.730	-0.824	-1.392	-3.019
	$_{HC}$Phe029	-7.605	-7.009	-0.001	0.011	-0.125	-0.482
His078	$_{HC}$Thr103	-7.529	-6.071	0.966	-1.110	-2.011	0.698
Ser117	$_{HC}$Gly056	-10.351	-14.699	10.015	-1.372	-3.135	-1.161
Tyr123	$_{HC}$Phe059	-4.465	-3.702	2.746	-1.375	-3.959	1.825
Arg125	$_{LC}$Ser096	-5.904	-9.773	0.057	-0.212	-0.479	4.502
	$_{LC}$Ser097	-3.552	-4.937	0.779	-0.378	-1.000	1.984

All energies are in kcal/mol.

The calculation has MP2/6-31G**/PCM level

The subscripts HC and LC indicate the heavy and light chain, respectively

[a] The interactions were reported in ref 30

[b] The interactions were shifted by HOP fragmentation

Table S9 The PIEDA of KN035/PD-L1 complex

PD-L1	KN035	PIE	ΔE^{es}	ΔE^{ex}	ΔE^{ct+mix}	ΔE^{di}	ΔG^{sol}
Tyr056[a]	Phe101	-7.357	-3.222	2.993	-2.269	-4.921	0.062
	Glu102	-4.224	-4.257	5.397	-1.490	-4.121	0.247
	Thr105	-3.508	-1.350	3.350	-1.643	-3.790	-0.075
Glu058[a]	Arg032	-54.069	-55.113	-0.001	0.116	-0.113	1.043
	Ser100	-29.974	-29.664	9.014	-4.309	-4.036	-0.978
	Gln116	-11.104	-9.490	-0.001	-0.083	-0.032	-1.497
Glu060	Lys027	-28.678	-58.255	0.009	-0.174	-0.295	30.038
Asp061[a]	Lys027	-76.952	-92.245	1.490	-2.476	-2.550	18.828
	Ser029	-30.562	-30.845	10.202	-5.592	-4.737	0.410
	Ser030	-11.927	-9.750	9.522	-5.228	-6.413	-0.058
	Arg032	-48.809	-47.853	0.401	-0.486	-1.076	0.205
	Ser100	-10.445	-8.297	0.724	-0.778	-1.466	-0.629
Lys062	Glu102	-21.701	-36.539	-0.001	-0.034	-0.3	15.174
Asn063[a]	Glu102	-6.883	-6.194	0.957	-0.368	-1.529	0.251
Gln066[a]	Cys033	-5.239	-4.907	0.024	-0.061	-0.245	-0.051
	Pro104	-12.456	-9.744	3.396	-2.276	-4.048	0.216
	Thr105	-21.563	-19.144	7.206	-4.021	-5.352	-0.252
Arg113[a]	Asp099	-105.383	-105.168	7.643	-3.744	-5.163	1.048
	Phe115	-13.989	-13.316	0.036	-0.281	-0.517	0.089
Met115[a]	Phe101	-3.153	-1.548	3.017	-0.902	-3.652	-0.069
Ala121	Ser111	-3.465	-2.141	2.168	-0.893	-2.166	-0.434
Asp122	Gly113	-3.960	-3.675	2.006	-1.397	-1.342	0.448
Tyr123[a]	Ala114	-7.463	-5.773	0.763	-0.443	-2.068	0.059
	Gln116	-6.100	-2.926	2.741	-1.799	-4.239	0.123
Arg125[a]	Gln116	-15.042	-19.258	5.429	-3.211	-3.419	5.416

All energies are in kcal/mol.

The calculation has MP2/6-31G**/PCM level

The subscripts HC and LC indicate the heavy and light chain, respectively

[a] The residues were related with the mutagenesis data in ref 31 and 32

Table S10 Water-bridge interaction in KN035/PD-L1

PIE	PD-L1	Water molecules	KN035	PIE
-27.897	Asp122	^{A}HOH311	Ala114	-13.16
-5.198	Asp061	^{A}HOH312	Ser030	-9.996
-12.84	Asp061	^{A}HOH330	Ser030	-4.636
-19.469	Lys062	^{A}HOH341	Glu102	-23.736
-8.59	Asn063			
-28.689	Asp073	^{B}HOH303	Asp103	-3.576
			Pro104	-11.639
-6.428	Glu060	^{B}HOH326	Lys027	-17.026
-17.847	Asp061			
-6.586	Ser117	^{B}HOH331	Thr110	-3.944
			Ser111	-8.994
-18.332	Glu058	^{B}HOH339	Gln116	-3.154
-19.335	Asp061			
-3.833	Arg125			
-4.821	Glu058	^{B}HOH364	Gln116	-3.83
-8.207	Asp061			
-18.396	Arg125			

All energies are in kcal/mol.

The calculation has MP2/6-31G**/PCM level

The superscripts A and B indicate the chain arrangement

Table S11 The PIEDA of Atezolizumab/PD-L1 complex

PD-L1	Atezolizumab	PIE	ΔE^{es}	ΔE^{ex}	ΔE^{ct+mix}	ΔE^{di}	ΔG^{sol}
Ala018	$_{HC}$Pro102	-5.209	-1.187	0.534	-0.002	-1.020	-3.533
Asp049	$_{LC}$Gln027	-4.195	-4.528	-0.001	-0.081	-0.122	0.537
	$_{LC}$Tyr093	-29.320	-28.335	11.178	-5.322	-4.924	-1.918
Ala052	$_{LC}$Tyr093[a]	-5.769	-2.648	3.622	-2.275	-4.577	0.109
Leu053	$_{LC}$His094	-3.614	-3.375	0.023	-0.421	-0.666	0.824
Tyr056[c]	$_{HC}$Trp050[a]	-9.812	-9.131	5.270	-1.894	-3.930	-0.127
Glu058[c]	$_{HC}$Ser052	-34.429	-32.178	7.696	-5.379	-4.790	0.221
	$_{HC}$Tyr054[a]	-8.997	-6.030	1.360	-1.155	-2.441	-0.731
	$_{HC}$Gly055[a]	-21.617	-19.487	5.094	-2.920	-3.834	-0.470
	$_{HC}$Gly056	-9.195	-9.292	0.064	2.427	-0.809	-1.585
	$_{HC}$Ser057[a]	-33.139	-29.452	10.098	-5.691	-5.286	-2.807
Met059	$_{HC}$Tyr054	-4.631	-3.003	0.777	-0.865	-1.747	0.207
Glu060	$_{HC}$Gly056	-5.084	-5.029	0.074	0.890	-0.267	-0.751
Asp061	$_{HC}$Gly055[a]	-5.502	-5.801	12.517	-3.586	-4.066	-4.566
	$_{HC}$Thr074	-8.112	-11.015	0.056	-1.193	-0.808	4.848
Lys062	$_{HC}$Gly055	-6.840	-7.432	3.150	-1.302	-1.598	0.342
Asn063[c]	$_{HC}$Gly056	-5.207	-5.124	0.377	0.032	-1.415	0.924
	$_{HC}$Ser057[a]	-12.479	-15.781	9.753	-2.947	-3.942	0.438
Gln066[c]	$_{HC}$Ser057[a]	-4.553	-3.465	0.619	-0.861	-1.316	0.469
Val068	$_{HC}$Tyr059	-3.263	-1.213	2.686	-1.119	-3.964	0.346
His069	$_{LC}$Pro095	-11.058	-8.780	1.386	-1.170	-2.139	-0.355
Gln077	$_{HC}$Tyr059	-11.001	-9.372	1.590	-1.563	-2.392	0.736
	$_{HC}$Lys065	-33.633	-39.294	6.684	-3.337	-4.244	6.559
His078	$_{HC}$Lys065	-18.115	-25.412	0.908	-1.460	-1.786	9.636
Tyr112	$_{HC}$Tyr054[b]	-17.445	-17.439	6.963	-3.331	-3.654	0.016
Arg113[c]	$_{HC}$Asp031	-62.020	-65.728	1.319	-1.838	-2.714	6.941
	$_{HC}$Ser032	-8.355	-6.667	0.978	-1.028	-1.673	0.035
	$_{HC}$Trp033	-14.501	-14.536	0.160	-0.482	-0.772	1.129
	$_{HC}$Ser052	-9.513	-9.290	3.433	-1.792	-2.354	0.491
	$_{HC}$Trp101	-4.288	0.088	0.363	-0.356	-0.903	-3.480
Ser117	$_{HC}$Arg099	-3.278	-3.893	0.001	-0.029	-0.143	0.785
Tyr118	$_{LC}$Leu092	-4.116	-1.633	0.305	-0.631	-1.982	-0.176
Gly120	$_{HC}$Arg099	-27.659	-28.284	6.225	-2.403	-2.789	-0.408
	$_{LC}$Tyr091	-5.267	-3.283	1.696	-1.221	-2.372	-0.086
Ala121	$_{HC}$Pro102	-13.458	-14.378	7.438	-2.731	-3.213	-0.573
Tyr123[c]	$_{HC}$Asp031	-10.199	-9.238	3.483	-2.123	-4.218	1.897
	$_{HC}$Trp101[a]	-5.266	-1.211	2.009	-0.206	-5.760	-0.098
Arg125[c]	$_{HC}$Ser030[a]	-6.762	2.562	1.216	-0.819	-1.351	-8.369
	$_{HC}$Asp031[a]	-95.935	-119.479	13.434	-6.671	-7.169	23.951
	$_{HC}$Tyr054[a]	-7.929	-6.355	4.616	-1.383	-4.534	-0.273

All energies are in kcal/mol.

The calculation has MP2/6-31G**/PCM level

The subscripts HC and LC indicate the heavy and light chain, respectively

[a] The interactions were reported in ref 29

[b] The interactions were shifted by HOP fragmentation

[c] The residues were related with the mutagenesis data in ref 32

Table S12 The PIEDA of Durvalumab/PD-L1 complex

PD-L1	Durvalumab	PIE	ΔE^{es}	ΔE^{ex}	ΔE^{ct+mix}	ΔE^{di}	ΔG^{sol}
Asp026	$_{LC}$Arg028[a]	-87.394	-114.207	12.854	-7.556	-6.485	28.000
	$_{LC}$Ser031	-22.327	-27.668	11.723	-5.253	-5.803	4.674
	$_{LC}$Ser032	-9.871	-10.206	0.072	0.331	-0.437	0.369
Tyr056	$_{HC}$Glu057	-12.877	-10.052	1.342	-1.208	-2.581	-0.378
Glu058	$_{HC}$Lys052[a]	-95.929	-112.415	5.345	-4.342	-3.928	19.412
Asp061	$_{HC}$Arg031	-12.782	-41.241	-0.001	-0.025	-0.164	28.649
Val111	$_{HC}$Glu105[a]	-4.964	-4.742	1.121	-0.492	-1.846	0.995
Tyr112	$_{HC}$Phe103	-4.380	-3.010	2.333	-1.221	-2.393	-0.090
Arg113	$_{HC}$Trp033	-3.572	-1.764	4.542	-1.322	-4.213	-0.815
	$_{HC}$Glu057[a]	-114.459	-121.319	17.989	-6.842	-6.772	2.486
	$_{HC}$Phe103	-9.546	-6.855	6.929	-2.250	-8.091	0.721
	$_{HC}$Gly104	-3.526	-3.685	-0.001	0.048	-0.191	0.303
Met115	$_{HC}$Glu057	-6.531	-4.860	3.438	-1.169	-2.956	-0.984
Asp122	$_{LC}$Leu095	-16.827	-14.828	1.568	-1.798	-2.646	0.878
Tyr123	$_{HC}$Tyr059	-5.404	-2.431	3.999	-1.400	-5.298	-0.274
	$_{HC}$Glu099	-5.738	-5.811	-0.001	-0.119	-0.153	0.347
	$_{HC}$Gly104	-4.461	-4.191	0.005	-0.163	-0.299	0.186
Lys124	$_{LC}$Ser094[a]	-28.124	-26.646	3.919	-2.001	-4.384	0.988
	$_{LC}$Trp097[a]	-3.350	-3.567	-0.001	0.039	-0.179	0.358
Arg125	$_{HC}$Glu099	-52.597	-55.381	0.002	-0.039	-0.213	3.034
	$_{HC}$Gly104[b]	-33.965	-35.077	9.682	-4.077	-4.988	0.494
	$_{HC}$Glu105	-50.995	-54.760	0.356	-0.322	-1.932	5.662
	$_{LC}$Ser031	-10.241	-8.559	-0.001	0.079	-0.101	-1.659
	$_{LC}$Gly093[b]	-30.101	-31.300	7.137	-2.640	-3.263	-0.034
	$_{LC}$Ser094	-4.637	-3.854	-0.002	0.048	-0.210	-0.619
	$_{LC}$Trp097	-8.361	-7.222	2.715	-1.439	-2.856	0.441

All energies are in kcal/mol.

The calculation has MP2/6-31G**/PCM level

The subscripts HC and LC indicate the heavy and light chain, respectively

[a] The interactions were reported in ref 33

[b] The interactions were shifted by HOP fragmentation

Table S13 Water-bridge interaction in Durvalumab/PD-L1

PIE	PD-L1	Water molecules	Durvalumab	PIE
-3.159	Arg125	^{A}HOH203	$_{LC}$Ser030	-11.483
-14.556	Ile126		$_{LC}$Ser031	-9.109
-14.53	Arg125	^{A}HOH210	$_{LC}$Ser094	-12.337
-3.672	Ile126			
-12.793	Tyr123	^{B}HOH304	$_{HC}$Asn050	-4.83
			$_{HC}$Glu099	-20.675
			$_{HC}$Gly104	-8.627
-22.273	Asp122	^{C}HOH303	$_{LC}$Leu095	-8.978
-30.606	Lys124			
-6.352	Thr020	^{C}HOH317	$_{LC}$Pro096	-10.208
-23.817	Asp122			

All energies are in kcal/mol.

The calculation has MP2/6-31G**/PCM level

The subscripts HC and LC indicate the heavy and light chain, respectively

The superscripts A, B and C indicate the chain arrangement

Table S14 The PIEDA of peptide-57/PD-L1 complex

PD-L1	peptide-57	PIE	ΔE^{es}	ΔE^{ex}	ΔE^{ct+mix}	ΔE^{di}	ΔG^{sol}
Tyr056	Trp008	-3.903	-0.553	5.547	-1.895	-7.023	0.022
	Ser009	-6.171	-4.185	2.676	-1.578	-3.068	-0.016
Glu058	Ser009	-3.295	-1.093	0.001	0.036	-0.172	-2.066
Asn063	Ser007	-4.478	-4.128	0.280	-0.477	-0.858	0.706
	Ser009	-4.905	-3.013	0.852	-1.292	-1.697	0.245
Gln066	Trp008	-18.640	-13.473	10.281	-3.554	-11.636	-0.258
Glu071	Pro004	-7.045	-6.121	0.770	-1.715	-2.058	2.079
	His005	-40.893	-78.420	0.398	-2.560	-1.976	41.665
Glu072	His005	-14.366	-21.387	-0.001	0.006	-0.109	7.125
Asp073	His005	-82.717	-105.691	14.025	-9.399	-9.329	27.677
Arg113	Trp010	-9.359	0.367	5.576	-2.233	-8.563	-4.507
Met115	Trp010	-3.326	-1.390	1.019	-0.986	-2.327	0.359

All energies are in kcal/mol.

The calculation has MP2/6-31G**/PCM level

Table S15 Water-bridge interaction in peptide-57/PD-L1

PIE	PD-L1	Water molecules	peptide-57	PIE
-13.242	Phe067	HOH307	His005	-8.676
-5.549	Glu072			
-19.393	Asp061	HOH318	Trp010	-5.297
-3.532	Arg113			
-5.253	Asn063	HOH321	Ser007	-14.672
-9.895	Gln077			

All energies are in kcal/mol.

The calculation has MP2/6-31G**/PCM level

Table S16 The PIEDA of peptide-71/PD-L1 complex

PD-L1	peptide-71	PIE	ΔE^{es}	ΔE^{ex}	ΔE^{ct+mix}	ΔE^{di}	ΔG^{sol}
Val055	MEA007	-3.247	-1.636	0.569	-0.951	-1.373	0.143
Tyr056	Phe001	-9.233	-4.073	5.230	-2.375	-8.451	0.436
Glu058	Phe001	-55.134	-55.365	0.000	-0.144	-0.293	0.668
	Tyr011	-6.274	-2.001	3.457	-3.101	-3.943	-0.687
	Leu012	-31.744	-34.306	11.392	-3.802	-5.122	0.095
	CCS013	-35.629	-35.216	9.371	-4.809	-5.646	0.671
Asp061	Leu012	-8.941	-6.942	1.941	-1.816	-2.796	0.673
Lys062	Gly014	-3.577	-3.145	0.052	0.364	-0.328	-0.521
Asn063	CCS013	-3.931	-2.466	0.641	-0.413	-1.358	-0.336
Gln066	Phe001	-10.036	-7.639	1.463	-1.317	-2.596	0.053
	MEA002	-4.454	-3.363	1.364	-1.288	-1.676	0.509
	9KK003	-4.112	-2.962	1.193	-0.441	-1.760	-0.143
Arg113	Tyr011	-15.686	-14.798	0.361	-0.920	-1.573	1.244
Met115	MEA007	-4.719	-1.835	2.655	-1.258	-4.330	0.049
Tyr123	Trp010	-7.952	-1.796	3.923	-0.606	-8.653	-0.819

All energies are in kcal/mol.

The calculation has MP2/6-31G**/PCM level

Table S17 Water-bridge interaction in peptide-71/PD-L1

PIE	PD-L1	Water molecules	peptide-71	PIE
-13.707	Gln077	HOH364	Phe001	-3.355
-3.552	Gln066	HOH457	9KK003	-6.714
-4.827	Asp073			
-5.706	Arg113	HOH110	Tyr011	-11.846

All energies are in kcal/mol.

The calculation has MP2/6-31G**/PCM level

Table S18 The PIEDA Analysis on BMS-8 and two PD-L1 molecules

PD-L1	PIE	ΔE^{es}	ΔE^{ex}	ΔE^{ct+mix}	ΔE^{di}	ΔG^{sol}
$_A$Tyr056	-10.512	-5.231	12.414	-3.852	-13.491	-0.352
$_A$Glu058	-6.219	-3.061	0.000	0.000	0.000	-3.158
$_A$Gln066	-4.031	-5.186	12.295	-3.148	-8.633	0.641
$_A$Met115	-5.523	-2.245	5.396	-1.464	-7.308	0.097
$_A$Asp122	-4.074	-1.772	4.119	-1.684	-3.232	-1.505
$_B$Tyr056	-4.053	-1.477	4.710	-1.426	-6.050	0.189
$_B$Met115	-4.688	-2.455	6.769	-1.552	-7.407	-0.042
$_B$Ser117	-3.352	-0.892	2.229	-0.810	-3.437	-0.442
$_B$Asp122	-25.134	-17.279	12.530	-4.492	-10.753	-5.140
$_B$HOH206	-4.193	-3.003	3.781	-1.820	-2.742	-0.409

All energies are in kcal/mol.

The calculation has MP2/6-31G**/PCM level

The subscripts A and B indicate the chain arrangement

Table S19 The PIEDA Analysis on BMS-202 and two PD-L1 molecules

PD-L1	PIE	ΔE^{es}	ΔE^{ex}	ΔE^{ct+mix}	ΔE^{di}	ΔG^{sol}
$_A$Thr020	-6.180	-2.885	6.364	-1.709	-5.595	-2.355
$_A$Tyr056	-3.528	-2.153	5.017	-1.568	-5.171	0.347
$_A$Met115	-4.798	-0.957	2.333	-1.189	-4.570	-0.415
$_A$Ser117	-3.409	-0.456	0.997	-0.907	-2.681	-0.362
$_A$Asp122	-33.126	-18.872	10.319	-4.898	-13.027	-6.648
$_B$Tyr056	-10.521	-3.576	11.140	-3.860	-14.325	0.101
$_B$Glu058	-7.171	-3.051	-0.001	-0.134	-0.208	-3.778
$_B$Gln066	-3.770	-1.312	5.334	-1.635	-5.643	-0.514
$_B$Asp073	-4.692	0.492	0.000	0.000	0.000	-5.184
$_B$Met115	-5.777	-1.830	1.728	-1.070	-4.628	0.023
$_B$Asp122	-4.941	-1.569	0.613	-0.480	-1.460	-2.045
$_A$HOH301	-3.246	-1.665	3.774	-2.029	-3.466	0.140
$_A$HOH316	-3.469	-1.188	2.388	-2.320	-2.851	0.502
$_B$HOH325	-3.055	-1.381	1.589	-1.411	-1.954	0.103

All energies are in kcal/mol.

The calculation has MP2/6-31G**/PCM level

The subscripts A and B indicate the chain arrangement

Table S20 The PIEDA Analysis on BMS-37 and two PD-L1 molecules

PD-L1	PIE	ΔE^{es}	ΔE^{ex}	ΔE^{ct+mix}	ΔE^{di}	ΔG^{sol}
$_A$Thr020	-3.375	1.978	4.067	-1.936	-5.327	-2.156
$_A$Tyr056	-3.835	-0.714	2.897	-1.178	-5.024	0.185
$_A$Met115	-4.965	-1.579	4.263	-1.074	-6.427	-0.148
$_A$Asp122	-36.479	-25.253	15.802	-7.147	-15.666	-4.215
$_B$Tyr056	-13.008	-9.012	20.204	-5.678	-18.334	-0.188
$_B$Glu058	-5.005	-2.577	0.000	0.041	-0.265	-2.204
$_B$Gln066	-5.815	-7.442	13.698	-4.038	-7.920	-0.113
$_B$Met115	-5.603	-1.659	6.276	-2.345	-7.847	-0.027
$_B$Asp122	-4.362	-1.608	0.602	-0.548	-1.942	-0.865
$_A$HOH227	-4.102	-2.180	3.836	-2.604	-3.490	0.336

All energies are in kcal/mol.

The calculation has MP2/6-31G**/PCM level

The subscripts A and B indicate the chain arrangement

Table S21 The PIEDA Analysis on BMS-200 and two PD-L1 molecules

PD-L1	PIE	ΔE^{es}	ΔE^{ex}	ΔE^{ct+mix}	ΔE^{di}	ΔG^{sol}
$_{A}$Thr020	-3.858	0.257	1.436	-1.219	-2.801	-1.531
$_{A}$Tyr056	-4.045	-0.898	4.510	-1.927	-5.912	0.182
$_{A}$Met115	-6.344	-2.285	3.872	-1.454	-6.462	-0.016
$_{A}$Ser117	-4.022	-1.746	1.372	-0.642	-2.792	-0.213
$_{A}$Asp122	-23.541	-13.987	4.519	-3.678	-8.052	-2.344
$_{B}$Tyr056	-12.350	-2.506	7.543	-3.727	-13.440	-0.221
$_{B}$Glu058	-4.079	-3.023	0.000	0.000	0.000	-1.056
$_{B}$Gln066	-11.432	-12.819	13.371	-3.892	-7.797	-0.296
$_{B}$Met115	-3.650	-3.434	11.872	-2.474	-9.789	0.175
$_{B}$Ala121	-3.323	-1.267	2.890	-0.853	-3.949	-0.144
$_{B}$Asp122	-5.636	-2.311	3.485	-2.657	-4.683	0.530
$_{B}$Tyr123	-4.074	-1.158	1.087	0.472	-4.276	-0.198
$_{A}$HOH229	-3.982	-2.085	2.080	-2.201	-1.980	0.205
$_{A}$HOH250	-8.218	-5.701	1.456	-1.964	-2.549	0.540
$_{B}$HOH306	-5.941	-15.699	18.160	-4.789	-3.361	-0.252
$_{B}$HOH316	-5.479	-16.327	21.239	-6.231	-3.546	-0.613

All energies are in kcal/mol.

The calculation has MP2/6-31G**/PCM level

The subscripts A and B indicate the chain arrangement
